# Contracting Out National Immunization Program Does Not Improve Vaccination Rate Nor Socioeconomic Inequality: A Case Study of Seasonal Influenza Vaccination in South Korea

**DOI:** 10.3389/fpubh.2021.769176

**Published:** 2021-11-04

**Authors:** Daseul Moon, Saerom Kim, Myoung-Hee Kim, Dawoon Jeong, Hongjo Choi

**Affiliations:** ^1^People's Health Institute, Seoul, South Korea; ^2^Department of Public Health, Graduate School of Public Health, Seoul National University, Seoul, South Korea; ^3^Research Institute of Public Health, National Medical Center, Seoul, South Korea; ^4^Division of Health Policy, Research Center, Korean Institute of Tuberculosis, Cheongju, South Korea; ^5^Department of Preventive Medicine, Konyang University College of Medicine, Daejeon, South Korea

**Keywords:** privatization, contracting out, public health, immunization program, vaccination, socioeconomic position, gender, inequality

## Abstract

The objective of the present study was to investigate if the policy for contracting out the Korean influenza National Immunization Program (NIP) for individuals aged ≥ 65 years affects a reduction in vaccination inequality based on gender and socioeconomic position (SEP). In South Korea, initially only public health centers provided influenza vaccination for free; however, starting from the fall of 2015, the program was expanded to include private medical institutions. The policy was expected to improve overall vaccination rate and reduce its inequality, through improving access to vaccination. The present study analyzed how the gap in the vaccination rate changed between before and after contracting out. A multivariate logistic regression model stratified by gender and SEP of individuals aged ≥ 65 years was used. The study also analyzed changes in the unvaccinated rates between before and after contracting out based on an interrupted time series model. The gap in the unvaccinated rate based on SEP present prior to contracting out of the NIP for individuals aged ≥ 65 years did not decrease afterwards. In particular, the step changes were 0.94% (95% confidence interval [CI]: 0.00, 1.89) and 1.34% (95% CI: 1.17, 1.52) in men and women, respectively. In the pre-policy period, among women, the unvaccinated rate of the medical aid beneficiaries group was 1.22-fold higher (95% CI: 1.12, 1.32) than that of the health insurance beneficiaries, and the difference was not reduced post-policy implementation (odds ratio: 1.27, 95% CI: 1.20, 1.36). The findings of the study were that contracting out of the NIP was not effective in improving vaccination rate nor resolving vaccination inequality. Future studies should focus on identifying the mechanism of vaccination inequality and exploring measures for resolving such inequality.

## Introduction

Coronavirus disease (COVID-19) vaccination is currently underway, and improving the vaccination rate is a key strategy for achieving herd immunity. Herd immunity is necessary to overcome the current COVID-19 pandemic (1). Effective immunization, a high-priority public health strategy for preventing disease transmission, is subject to strict governmental control in terms of planning, procurement, and service provision. In particular, guaranteeing equal access without discrimination or exclusion of specific groups is important. However, the National Health Insurance system in South Korea (2–5) creates a barrier to health care access including vaccination. In Korea, majority of the population (97%) are covered by the National Health Insurance Services, while the most disadvantaged are covered by the Medical Aid Program. In theory, the entire population have healthcare coverage. However, the required copayment is considerable (about 65%), which still hampers access to healthcare by the poor. Moreover, private healthcare facilities account for 90% of all hospital beds. Although for-profit hospitals are not allowed in Korea, many private facilities are profit driven. Under the circumstances, among various accessibility dimensions, physical and economic accessibility could deteriorate. Improvement in physical and economic accessibility is achievable through policies guaranteeing free vaccination, as well as by securing enough medical institutions that can provide such a service within a reachable distance (6). In Korea, the National Immunization Program (NIP) was initially carried out mostly by public medical institutions. To increase immunization accessibility, the Korean government then pursued a policy for expanding the NIP to include private medical institutions (7) COVID-19 vaccination was carried out in a similar manner: in the early stages, vaccination was carried out through most public medical institutions and was subsequently expanded through the NIP to include private medical institutions.

In other words, the government has contracted out the NIP from the public sector to the private sector. With “contracting out,” private entities are contracted to provide public services that used to be provided directly by the government. Expansion of the immunization program to include private medical institutions is one approach by which the Korean government is “contracting out” as part of the NIP. The Korean government explains this as a public–private partnership with private medical institutions instead of “contracting out.” However, the authors believe that understanding this concept as contracting out would be more appropriate. The contracting government plays the role of the purchaser and manager, and the contracted private medical institution plays the role of an immunization service provider based on a contract. The parties do not share finance, management, nor risks in the process of providing the immunization services (8, 9). On the one hand, given that privatization refers to all efforts to introduce market mechanisms to public service delivery by public-to-private transfer of authority over ownership, management, finance, and/or control (8, 10), contracting out the NIP could be conceptualized and considered as one type of privatization (8, 11, 12).

The goals of contracting out of the NIP are to improve the accessibility and convenience of immunization, which are consistent with the motivation for privatization of general public services. Privatization, especially the need for and implementation of contracting out, is based on the belief that private entities are able to deliver services more effectively and efficiently than public entities (12, 13). Contracting out offers the following benefits: (1) there is no need to increase the number of public servants to directly provide the service, which can help reduce government expenditure; (2) the expertise, resources, and technology of private institutions could be utilized, while the quality of service could be improved by promoting competition among private institutions by giving more choices to the users; and (3) the service delivery practice of contracted private institutions could be assessed more objectively than in the case of the government assessing its own practice. Therefore, greater emphasis is placed on the accountability of the private institutions, meaning the service providers. This could allow for more effective and efficient service delivery (14, 15). However, it is uncertain whether contracting out could actually produce such outcomes. In fact, it may produce inefficient outcomes: management and supervision costs are incurred, government accountability and public interest may be compromised, and cost savings based on reduced manpower and lower wages by private contractors could actually lead to a decline in the quality of service. For example, when the British National Health Service contracted out its cleaning service, a reduction in cleaning staff for efficiency led to an increased risk of hospital-acquired infection (16). However, empirical review and discussions on the effects of contracting out of essential public health services, not “non-essential” services, are still lacking.

South Korea expanded its influenza vaccination program for individuals aged ≥ 65 years to include private medical institutions. The advocates of the policy have claimed that it can contribute to improving the overall vaccination rate and addressing the vaccination inequality with the purpose of improving immunization accessibilities. Consequently, the proportion of vaccinations carried out in private medical institutions increased. However, the proportion of vaccinations carried out in public health centers and other public institutions decreased. Given this evidence, a previous study has reported that contracting out vaccination services does not contribute to an overall improvement in the vaccination rate (17). The present study goes a step further to identify the influence of such contracting out on gender and socioeconomic inequality in immunization. The study investigated gender-differences in the effects of contracting out of influenza vaccination for individuals aged ≥ 65 years and whether contracting out contributed to reducing the gap in vaccination rate based on socioeconomic position (SEP).

## Materials and Methods

### National Immunization Program on Seasonal Influenza in South Korea

The influenza NIP in South Korea began in 1997 as a pilot program for individuals aged ≥ 65 years and patients with cardiopulmonary disease. Free vaccination for individuals aged ≥ 65 years which began in 2005 at public health centers, was not for free at private medical institutions. Therefore, out-of-pocket expenditure was incurred when individuals were vaccinated at private medical institutions. After the program was subsequently expanded to include private medical institutions starting from the 2015–2016 flu season (17), no one paid expenses for the vaccination anymore.

### Study Population

The present study used 2013–2019 data from the Korean Community Health Survey (KCHS) (18).

KCHS, conducted annually by the Korean Disease Control and Prevention Agency since 2008, has participation from approximately 250 public health centers throughout South Korea. The KCHS uses two rounds of systematic sampling (individuals aged ≥ 19 years) to collect nationally representative data from surveying ~2,20,000 individuals each year. The present study selected individuals aged ≥ 65 years at the time of the survey and included their “Yes” or “No” responses to whether they received annual influenza vaccination. Data from 2015, the year when contracting out of the vaccination service was implemented, were excluded since it could cause confusion in the analysis. However, the vaccination rate among the 2015 survey participants was calculated separately for subsequent calculation of the annual vaccination rate. Lastly, participants who did not respond to questions on covariates were also excluded.

### Measurement

The two major independent variables were the survey participation time point and medical aid beneficiary status, as a proxy indicator of SEP. For the survey participation time point, 2013 and 2014 survey participants were defined as the pre-policy implementation group (pre-group) and 2016–2019 participants were defined as the post-policy implementation group (post-group). South Korea operates a national health insurance system. While 97% of the population is covered under this system, the remaining 3% receives healthcare coverage through a tax-based system called medical aid. The medical aid beneficiaries represent the poor and socially deprived population who qualify based on the following conditions: (1) earn ≤ 40% of the standard median income; (2) inability to work due to health issues, disabilities, etc.; and (3) have no dependents (or support family). Individuals who indicated that they received medical aid benefits during the year covered by the KCHS were defined as medical aid beneficiaries, while all others were categorized as health insurance beneficiaries. The outcome variable was defined as responding “Yes” to the question “Have you received an influenza vaccination in the past year?” To analyze the changes in annual vaccination rate, the gender-stratified total vaccination rate among individuals aged ≥ 65 years for the survey year and the vaccination rate based on SEP after gender stratification were measured separately. Lastly, among the variables known to influence healthcare utilization, marital status, region (residence), recent labor (paid/unpaid) experience, monthly household income, smoking history, alcohol consumption history, hypertension, and diabetes mellitus (DM) were measured as covariates.

### Statistical Analysis

Participants in the 2015 survey were excluded from all analyses, except for the trend analysis on changes in annual vaccination rate. All analyses were performed separately by gender. For the vaccination rate based on the type of health coverage, differences in the distribution of baseline variables were tested using Pearson's chi-square test. To analyze the differences based on privatization policy and health coverage together, a multivariate logistic regression model stratified for policy and health coverage types was constructed. With the pre-policy implementation health insurance beneficiaries as the reference group, the odds ratios (ORs) of the unvaccinated rates for influenza in the pre-policy implementation medical aid beneficiaries, post-policy implementation health insurance beneficiaries, and post-policy implementation medical aid beneficiaries were calculated. The gap in the unvaccinated rate based on health coverage type before and after the implementation of the policy was examined separately. Changes in the unvaccinated rate between before and after the implementation of the policy were analyzed for the health insurance subscribers and medical aid beneficiaries. The additive interaction of health coverage type and policy implementation was measured by the relative excess risk due to interaction and the proportion of disease attributable to interaction. To analyze the time-series changes in the vaccination rate between before and after the implementation of the policy, an interrupted time-series model was constructed as shown below. We considered that a level change and slope change model would be appropriate to identify the impact of contracting out of the NIP, because the total number of hospitals providing free vaccination was immediately increased right after the policy change and the change could gradually change the health seeking behaviors of people (19). The model below included participants in the 2015 survey, which was conducted between August 31st and November 8th, which overlaps with the period when privatization of influenza vaccination was fully implemented. Therefore, it was assumed that the effect of the implementation of the policy would appear starting from the 2016 survey.


(1) $$\begin{array}{r} Y_{t}=\beta_{0}+\beta_{1}T+\beta_{2}X_{1}+\gamma_{1}TX_{1}+\delta C^{t-1} \end{array}$$

*Y*_*t*_ is the unvaccinated rate at year t. *T* is year t. *X*_1_ is an indicator of the introduction of a new policy (after the policy is introduced, *X*_1_ = 1; otherwise, 0). β_1_ is the slope before the new policy, β_2_ is a step-down after the policy was introduced and γ_1_ indicates the interaction of *X*_1_ and *T*. δ and C are a matrix of potential confounders in t−1 year and their coefficients. With seven time points, we considered a potential confounder that was the log transformed influenza incidence in t−1 year, because the incidence in t−1 year would affect the health behaviors of people in t year. For the sensitivity analysis, we also constructed a slope change model as the simplest model with different lags, with assumption that the policy change could not affect the level change. We conduct the Cumby-Huizinga test to identify appropriate lags in the models. STATA/SE version 15 (StataCorp LLC, College Station, TX, USA) was used for all statistical analyses.

## Results

### Baseline Characteristics

A total of 4,58,804 individuals aged ≥ 65 years participated in the surveys. Of these, 3,94,284 were included in the final analysis after excluding participants in the 2015 survey (*n* = 63,141) and individuals who did not provide a response for immunization status (n = 1), marital status (*n* = 136), smoking status (*n* = 23), alcohol consumption status (*n* = 92), and DM status (*n* = 1,127). Among the female health insurance subscribers, the unvaccinated rate was highest in 2013 (14.8%), and it decreased to 8.6% in 2019. Among the female medical aid recipients, the unvaccinated rates in 2013 and 2019 were 17.7 and 12.4%, respectively (Table 1). Among the male health insurance subscribers, the unvaccinated rates were highest (16.8%) and lowest (11.2%) in 2013 and 2019, respectively. Among the male medical aid recipients, the unvaccinated rates in 2013 and 2019 were 22.1 and 15.1%, respectively (Table 2). Among the overall study population, the unvaccinated rate was lowest among those aged 75–84 years, living with a partner, living in a rural region, and drinking less. Participants under hypertension and DM treatment had lower unvaccinated rates than those under no such treatment among both men and women.

**Table 1:** Vaccination rates among female participants based on different baseline characteristics stratified by type of health coverage.

		**Health insurance beneficiaries (*****n*** **=** **215,541)**					**Medical aid beneficiaries (*****n*** **=** **16,140)**				
		**Vaccinated**		**Unvaccinated**		***P*-value**	**Vaccinated**		**Unvaccinated**		***P*-value**
		**n**	**%**	**n**	**%**		**n**	**%**	**n**	**%**	
Age (years)	65–74	1,00,277	87.7	14,105	12.3	<0.001	5,717	85.1	1,004	14.9	<0.001
	75–84	76,837	91.4	7,200	8.6		6,512	88.0	887	12.0	
	≥ 85	14,471	84.5	2,651	15.5		1,680	83.2	340	16.8	
Marital status	Living with partner	91,952	89.5	10,747	10.5	<0.001	3,191	87.3	463	12.7	0.001
	Divorced	1,952	82.3	421	17.7		830	82.6	175	17.4	
	Widowed	95,682	88.6	12,339	11.4		9,390	86.3	1,496	13.7	
	Separated	1,542	82.6	324	17.4		157	86.3	25	13.7	
	Single	457	78.5	125	21.5		341	82.6	72	17.4	
Region	Urban	70,695	87.9	9,755	12.1	<0.001	6,052	85.7	1,012	14.3	0.102
	Rural	1,20,890	89.5	14,201	10.5		7,857	86.6	1,219	13.4	
Recent labor experiences	No	1,29,297	88.8	16,338	11.2	0.026	12,117	86.1	1,952	13.9	0.620
	Yes	62,288	89.1	7,618	10.9		1,792	86.5	279	13.5	
Household income (month)	<5,00,000 (KRW)	47,117	89.0	5,832	11.0	<0.001	5,892	85.2	1,023	14.8	0.017
	5,00,000–9,99,999	48,436	89.8	5,490	10.2		4,774	86.7	730	13.3	
	10,00,000–19,99,999	41,021	88.6	5,285	11.4		1,186	85.9	194	14.1	
	20,00,000–29,99,999	30,112	89.2	3,643	10.8		1,755	87.9	241	12.1	
	30,00,000–39,99,999	9,642	86.7	1,480	13.3		114	85.1	20	14.9	
	≥ 40,00,000	15,257	87.3	2,226	12.7		188	89.1	23	10.9	
Smoking history	Never smoked	1,83,860	89.1	22,530	10.9	<0.001	12,287	86.5	1,914	13.5	<0.001
	Previously smoked	4,388	86.9	664	13.1		836	87.4	121	12.6	
	Current smoker (occasionally)	457	80.9	108	19.1		108	85.0	19	15.0	
	Current smoker (daily)	2,880	81.5	654	18.5		678	79.3	177	20.7	
Alcohol consumption	No	1,32,473	88.9	16,499	11.1	<0.001	10,706	86.2	1,719	13.8	0.067
	Fewer than once a month	31,290	89.6	3,619	10.4		1,602	87.4	231	12.6	
	Once a month	9,510	89.4	1,131	10.6		489	85.8	81	14.2	
	2–4 times/month	10,664	87.9	1,474	12.1		665	86.4	105	13.6	
	2–3 times/week	4,484	86.6	696	13.4		246	84.2	46	15.8	
	≥ 4 times/week	3,164	85.5	537	14.5		201	80.4	49	19.6	
Hypertension	No	79,397	86.1	12,852	13.9	<0.001	4,911	82.6	1,033	17.4	<0.001
	On treatment	1,09,014	91.2	10,460	8.8		8,728	88.4	1,142	11.6	
	Diagnosed without treatment	3,174	83.1	644	16.9	<0.001	270	82.8	56	17.2	<0.001
Diabetes mellitus	No	1,52,874	88.4	20,095	11.6		10,279	85.2	1,785	14.8	
	On treatment	36,504	91.2	3,540	8.8		3,430	89.3	413	10.7	
	Diagnosed without treatment	2,207	87.3	321	12.7		200	85.8	33	14.2	
Survey year	2013	27,403	85.2	4,761	14.8	<0.001	1,973	82.3	425	17.7	<0.001
	2014	27,637	85.6	4,653	14.4		2,287	83.3	457	16.7	
	2016	30,801	89.2	3,714	10.8		2,331	85.7	389	14.3	
	2017	32,931	90.1	3,635	9.9		2,319	88.4	304	11.6	
	2018	35,743	90.6	3,689	9.4		2,594	89.2	315	10.8	
	2019	37,070	91.4	3,504	8.6		2,405	87.6	341	12.4	

**Table 2:** Vaccination rates among male participants based on different baseline characteristics stratified by type of health coverage.

		**Health insurance beneficiaries (*****n*** **=** **155,125)**					**Medical aid beneficiaries (*****n*** **=** **7,478)**				
		**Vaccinated**		**Unvaccinated**		***P*-value**	**Vaccinated**		**Unvaccinated**		***P*-value**
		**n**	**%**	**n**	**%**		**n**	**%**	**n**	**%**	
Age (years)	65–74	76,858	83.5	15,204	16.5	<0.001	2,943	77.6	850	22.4	<0.001
	75–84	50,554	91.3	4,842	8.7		2,714	86.7	417	13.3	
	≥ 85	6,678	87.1	989	12.9		466	84.1	88	15.9	
Marital status	Living with partner	1,19,391	87.0	17,776	13.0	<0.001	3,690	84.9	658	15.1	<0.001
	Divorced	2,068	73.9	731	26.1		790	77.1	235	22.9	
	Widowed	10,559	84.8	1,899	15.2		1,240	80.2	307	19.8	
	Separated	1,823	77.9	516	22.1		167	74.6	57	25.4	
	Single	249	68.8	113	31.2		236	70.7	98	29.3	
Region	Urban	52,337	85.1	9,152	14.9	<0.001	2,895	81.7	650	18.3	0.645
	Rural	81,753	87.3	11,883	12.7		3,228	82.1	705	17.9	
Recent labor experiences	No	67,810	87.1	10,060	12.9	<0.001	4,998	82.0	1,098	18.0	0.611
	Yes	66,280	85.8	10,975	14.2		1,125	81.4	257	18.6	
Household income (month)	<500,000 (KRW)	21,789	87.1	3,235	12.9	<0.001	1,903	79.6	489	20.4	0.002
	5,00,000–9,99,999	32,989	88.0	4,514	12.0		2,543	82.5	538	17.5	
	10,00,000–19,99,999	37,581	86.5	5,870	13.5		700	82.4	150	17.6	
	20,00,000–29,99,999	22,612	85.9	3,724	14.1		808	85.0	143	15.0	
	30,00,000–39,99,999	8,197	83.9	1,569	16.1		86	87.8	12	12.2	
	≥ 40,00,000	10,922	83.7	2,123	16.3	<0.001	83	78.3	23	21.7	<0.001
Smoking history	Never smoked	29,790	86.7	4,552	13.3		1,171	82.0	257	18.0	
	Previously smoked	79,643	88.6	10,281	11.4		3,302	85.4	566	14.6	
	Current smoker (occasionally)	1,955	83.3	393	16.7		112	76.2	35	23.8	
	Current smoker (daily)	22,702	79.6	5,809	20.4		1,538	75.6	497	24.4	
Alcohol consumption	No	55,704	87.5	7,955	12.5	<0.001	3,266	82.8	678	17.2	0.027
	Fewer than once a month	13,061	88.0	1,781	12.0		536	83.8	104	16.3	
	Once a month	7,295	87.4	1,052	12.6		242	80.9	57	19.1	
	2–4 times/month	18,070	86.7	2,762	13.3		632	81.5	143	18.5	
	2–3 times/week	16,461	85.3	2,832	14.7		583	81.0	137	19.0	
	≥ 4 times/week	23,499	83.5	4,653	16.5		864	78.5	236	21.5	
Hypertension	No	65,860	83.7	12,832	16.3	<0.001	2,832	78.8	764	21.2	<0.001
	On treatment	65,120	89.7	7,481	10.3		3,103	85.1	545	14.9	
	Diagnosed without treatment	3,110	81.2	722	18.8		188	80.3	46	19.7	
Diabetes mellitus	No	1,04,869	85.6	17,578	14.4	<0.001	4,581	81.0	1,072	19.0	<0.001
	On treatment	27,508	89.8	3,134	10.2		1,447	85.3	250	14.7	
	Diagnosed without treatment	1,713	84.1	323	15.9		95	74.2	33	25.8	
Survey year	2013	19,343	83.2	3,901	16.8	<0.001	802	77.9	228	22.1	<0.001
	2014	19,364	83.1	3,930	16.9		933	78.9	249	21.1	
	2016	21,272	86.2	3,396	13.8		992	82.2	215	17.8	
	2017	23,066	87.9	3,174	12.1		1,099	83.6	215	16.4	
	2018	24,855	88.2	3,326	11.8		1,152	82.5	244	17.5	
	2019	26,190	88.8	3,308	11.2		1,145	84.9	204	15.1	

### Interaction Between Socioeconomic Position and Privatization

A stratified multivariate analysis of the changes in the unvaccinated rate based on SEP before and after the implementation of the policy was conducted. Among women, the unvaccinated rate of the pre-policy implementation health insurance beneficiaries (reference group) was 14.6%, while that of the medical aid beneficiaries was approximately 1.22-fold higher (17.2%; 95% CI: 1.12, 1.32). The unvaccinated rate of the post-policy implementation medical aid beneficiaries was ~1.27-fold (95% CI: 1.20, 1.36) higher than that of the health insurance subscribers. Both health insurance subscribers and medical aid beneficiaries showed a decrease in the unvaccinated rate between before and after the implementation of the policy, with ORs of 0.63 (95% CI: 0.61, 0.65) and 0.69 (95% CI: 0.63, 0.76), respectively. No interaction was found between the privatization policy and SEP. The pre-policy implementation SEP-based gap in the unvaccinated rate was maintained after policy implementation (Table 3). Similar results were found among the men (Table 4).

**Table 3:** Contracting out program-stratified association of socioeconomic position with the unvaccinated rate for seasonal influenza among women.

	**Seasonal influenza vaccination program (*****n*** **=** **2,31,681)**				**OR (95% CI) for the contracting out within strata of the type of health coverage**
	**Before contracting out (2013–2014)**		**After contracting out (2016–2019)**		
	**Vaccinated (%)/aannggeellUnvaccinated (%)**	**OR (95% CI)**	**Vaccinated (%)/aannggeellUnvaccinated (%)**	**OR (95% CI)**	
Type of health coverage					
Health insurance beneficiaries	55,040 (85.4)/aannggeell9,414 (14.6)	reference	136,545 (90.4)/aannggeell14,542 (9.6)	0.63 (0.61, 0.65)aannggeell *P* <0.001	0.63 (0.61, 0.65)aannggeell *P* <0.001
Medical aid beneficiaries	4,260 (82.9)/aannggeell882 (17.2)	1.19 (1.10, 1.29)aannggeell *P* <0.001	9,649 (87.7)/aannggeell1,349 (12.3)	0.80 (0.75, 0.86)aannggeell *P* <0.001	0.69 (0.63, 0.76)aannggeell *P* <0.001
OR (95% CI) for the type of health coverage within strata of the contracting out		1.22 (1.12, 1.32)aannggeell *P* <0.001		1.27 (1.20, 1.36)aannggeell *P* <0.001	

*Measure of interaction on additive scale: RERI (95% CI) = −0.02 (−0.12, 0.08); AP (95% CI) = −0.02 (−0.15, 0.11)*.

*Measure of interaction on multiplicative scale: ratio of ORs (95% CI) = 1.07 (0.97, 1.18)*.

*ORs are adjusted for age, marital status, region, recent labor experiences, household income, smoking history, alcohol consumption, hypertension, and diabetes mellitus*.

*RERI, relative excess risk due to interaction; CI, confidence interval; AP, proportion attributable to interaction; OR, odds ratio*.

**Table 4:** Contracting out program-stratified association of socioeconomic position with the unvaccinated rate for seasonal influenza among men.

	**Seasonal influenza vaccination program (*****n*** **=** **162,603)**				**OR (95% CI) for the contracting out within strata of the type of health coverage**
	**Before contracting out (2013–2014)**		**After contracting out (2016–2019)**		
	**Vaccinated (%)/aannggeellUnvaccinated (%)**	**OR (95% CI)**	**Vaccinated (%)/aannggeellUnvaccinated (%)**	**Vaccinated (%)/aannggeellUnvaccinated (%)**	
Type of health coverage					
Health insurance beneficiaries	38,707 (83.2)/aannggeell7,831 (16.8)	reference	95,383 (87.8)/aannggeell13,204 (12.2)	0.72 (0.70, 0.75)aannggeell *P* <0.001	0.72 (0.70, 0.75)aannggeell *P* <0.001
Medical aid beneficiaries	1,735 (78.4)/aannggeell477 (21.6)	1.23 (1.10, 1.37)aannggeell *P* <0.001	4,388 (83.3)/aannggeell878 (16.7)	0.90 (0.83, 0.98)aannggeell *P* = 0.017	0.77 (0.67, 0.88)aannggeell *P* <0.001
OR (95% CI) for the type of health coverage within strata of the contracting out		1.23 (1.10, 1.38)aannggeell *P* <0.001		1.26 (1.16, 1.36)aannggeell *P* <0.001	

*Measure of interaction on additive scale: RERI (95% CI) = −0.08 (−0.23, 0.07); AP (95% CI) = −0.09 (-0.26, 0.08)*.

*Measure of interaction on multiplicative scale: ratio of ORs (95% CI) = 0.99 (0.87, 1.13)*.

*ORs are adjusted for age, marital status, region, recent labor experiences, household income, smoking history, alcohol consumption, hypertension, and diabetes mellitus*.

*RERI, relative excess risk due to interaction; CI, confidence interval; AP, proportion attributable to interaction; OR, odds ratio*.

### Time-Serial Trends of the Unvaccinated Rate

**Figure 1:**
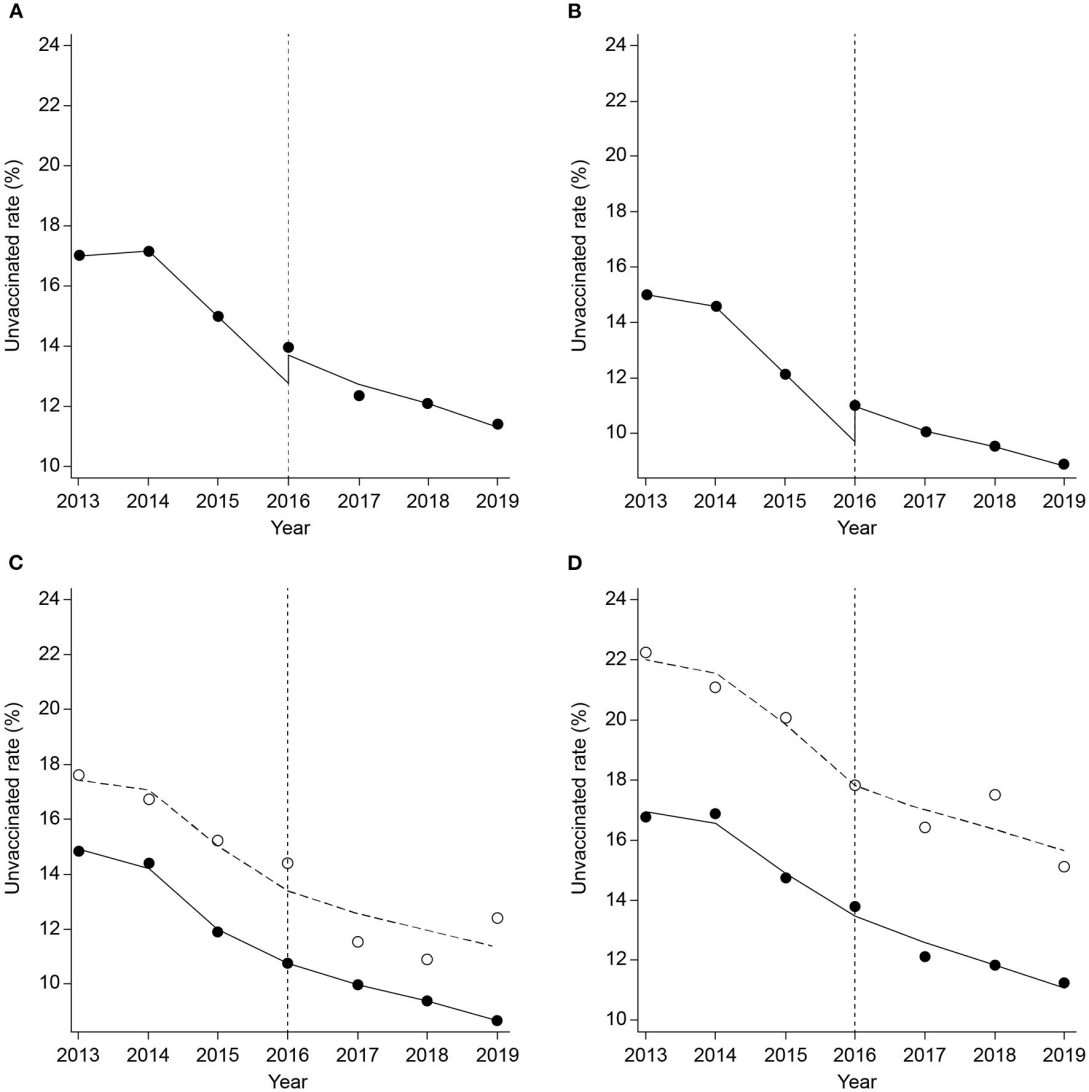
Change in the unvaccinated rate before and after the contracting out of the National Immunization Program [**(A)**: male; **(B)**: female; **(C)**: male by the type of health coverage; **(D)**: female by the type of health coverage; **(C,D)**: solid line indicates health insurance beneficiaries and dashed line indicates medical aid beneficiaries].

In the gender-stratified analysis, there was a decrease in the unvaccinated rate from before to after the implementation of the policy among both men and women. However, changes in the decreasing trend slope were not affected by the implementation of the policy, and there was no level change in the unvaccinated rate (Figure 1). In the detailed analysis, the slope change of the unvaccinated rates, which was adversely increased after the implementation of the policy among men (lag 0) and women (lag 3) were 0.94% (95% CI: 0.00, 1.89) and 1.34% (95% CI: 1.17, 1.52), respectively. In the analysis based on health coverage type, there was a 1.40% increase in the unvaccinated rate after the implementation of the policy (95% CI: 1.34, 1.46) among male health insurance beneficiaries (lag 3) with a level change (coefficient: 0.86, 95% CI: 0.78, 0.94). We mainly present the level and slope change model and additionally describe the results from the slope change model in Table 5. There were little differences between the models.

**Table 5:** Percent point changes in unvaccinated rates by gender and type of health coverage.

**Gender**	**Variables**	**Change of percent point**	**95% CI**	
Men		*T*	−1.86	−2.37, −1.35
		*X_1_*	0.39	−1.62, 2.39
		T X_1_^*^	0.94	−0.00, 1.89
Women (lag 3)		*T*	−2.15	−2.21, −2.09
		*X_1_*	0.77	0.54, 1.00
		T X_1_^**^	1.34	1.17, 1.52
Men	Health insurance	*T*	−2.20	−2.22, −2.17
	beneficiaries (lag 3)	*X_1_*	0.86	0.78, 0.94
		*T X_1_^†^*	1.40	1.34, 1.46
	Medical aid	*T*	−1.58	−2.69, −0.47
	beneficiaries (lag 3)	*X_1_*	−0.33	−4.62, 3.96
		*T X_1_*	0.86	−2.30, 4.02
Women	Health insurance	*T*	−1.89	−2.40, −1.39
	beneficiaries	*X_1_*	0.46	−1.62, 2.54
		*T X_1_*	0.97	−0.02, 1.96
	Medical aid	*T*	−1.13	−1.97, −0.28
	beneficiaries (lag 1)	*X_1_*	−1.18	−3.03, 0.68
		*T X_1_*	0.41	−1.51, 2.34

*CI, confidential interval*,

* *slope change model: –0.92 (–1.72, –0.12)*,

** *slope change model: –0.81 (–0.96, –0.65)*,

† *slope change model: –0.80 (–0.85,– 0.75)*.

## Discussion

The findings of the present study confirmed that privatization of the influenza NIP did not reduce SEP-based vaccination inequality. Analyses based on gender and SEP showed an improvement effect on the vaccination rate among female medical aid recipients. However, the results did not show privatization of the NIP as having an overall improvement effect on the vaccination rate among the general population. These findings are consistent with those of a recent South Korean study that analyzed different data sources (17). This study presented policy implementation without consideration for fundamental causes of vaccine hesitancy as one of the factors. Privatization of the NIP had little effect on improving the vaccination rate and did not reduce SEP-based vaccination inequality. This may reflect the need for more active consideration of social determinants in the NIP. Safety awareness, the gap in information access due to health literacy, the gap between urban and rural regions, financial gap, and differences in perception of authoritative government policies have been presented as social determinants of vaccine hesitancy (6).

The gender-based difference in vaccination is also important. Studies outside South Korea have reported that women generally have higher vaccine hesitancy than men (20, 21). Consequently, men have a higher influenza vaccination rate (22–25). However, in South Korea, elderly women have a higher vaccination rate, with the gender-based gap appearing especially among young-old women and men. This may be related to young-old women, aged 65–74 years, having higher outpatient service utilization rates than their male counterparts (26, 27). Moreover, a higher percentage of young-old women may have a regular source of care (28). There is a higher likelihood of women being informed about receiving free influenza vaccination at their regular outpatient medical institutions and acting on such information to actually get vaccinated. Such a phenomenon also appears to be related to the free vaccination service offered at private medical institutions as part of the public service offered by the government. The service should be accepted as a welfare service, rather than other preventive services that have out-of-pocket costs. Elderly Korean women represent the group with the lowest income among all age and gender groups (29). It is suspected that the higher vaccination rate may be related to the elimination of out-of-pocket costs for influenza vaccination at private medical institutions. These costs used to amount to 20,000–40,000 won for the poorest group before the implementation of the NIP and is still charged to the people under the age of 65. It can be viewed that the policy had a marginal benefit in this group, and could explain the largest improvement effect among female medical aid recipients after the implementation of the policy. Such a tendency might be consistent with existing evidence that service use increases among the poorest and women when public spending is increased (30, 31). However, an increase in public spending in such cases involves a concept that does not differentiate between the public or private status of the service provider. Moreover, it does not consider the contextual influence associated with how the service is provided. Therefore, it is only partially valid in explaining the policy effect in the present study.

The findings of the present study were somewhat different from those of other international academic studies on the effects of contracting out influenza vaccination. An overseas literature review evaluated the effectiveness of contracting out primary healthcare services, including vaccination, in low- and middle-income countries. The study reported that contracting out led to improved service accessibility through an expanded range of service provision, utilization, and coverage (32). A Korean study that assessed the effects of contracting out vaccination reported that contracting out increased accessibility, whereby the vaccination rate among individuals aged ≥ 65 years improved (33). However, in the present study, which conducted an analysis with consideration of the time trend, there was no significant change in increasing trend in the vaccination rate between before and after the implementation of contracting out. In other words, the increase in the vaccination rate reflected the increasing trend that presents before contracting out; therefore, it would be difficult to claim that such an effect was a result of contracting out. Meanwhile, just as in the study by Liu et al. (32), contracting out was unable to reduce SEP-based vaccination inequality. Even after contracting out, differences in influenza vaccination among health insurance subscribers and medical aid recipients remained.

Contracting out as a privatization approach does not reduce SEP-based inequality, but is likely to exacerbate it (34). The generation of a new public service user fee, an increase in the existing user fee, privatization of the social safety net, a reduction in wages and benefits, and socioeconomic segregation are the five mechanisms discussed. Influenza vaccination for the elderly Korean population adopted a privatization strategy in the form of contracting out, which is free of charge and targets the entire population aged ≥ 65 years. Therefore, instead of the first four mechanisms, the last mechanism appears to be applicable. In other words, contracting out influenza vaccination did not consider the characteristics of individuals aged ≥ 65 years nor did it include resolution of inequality as one of its goals from the beginning. Therefore, such results could be attributable to the absence of accountability and a strategy for such factors. Quantitative expansion of vaccination institutions could be a strategy that does not sufficiently consider the characteristics of the elderly population. It also does not consider the difference in accessibility by stages of healthcare use based on the SEP of the vaccination “customers” (35). The low-income class has a high likelihood of not receiving vaccination due to the burden of indirect costs, including transportation fee for going to the medical institution. However, economic accessibility from that perspective has not been considered. The need for vaccination and access to relevant information may vary depending on income level; however, such factors have not been considered. Kim et al. (33) examined the factors that influence influenza vaccination sites. They found that, among individuals aged ≥ 65 years, the OR for receiving vaccination at a public health center was significantly higher in the fourth income quartile than in the first income quartile. However, contracting out only has the goal of expanding vaccination to private medical institutions, rather than seeking the role of public health centers. Contracted providers need to only provide immunization services for vaccines requested according to the terms of the contract; thus, they have no incentive to provide services by identifying or prioritizing vulnerable populations, such as the elderly and impoverished. Therefore, contracting out vaccination by the government with the goal and strategy of only improving physical accessibility through quantitative expansion of vaccination sites could be understood as maintaining the same vaccination rate and socioeconomic inequalities affecting the vaccination rate.

Healthcare services, as public goods with non-exclusionary and non-competitive characteristics, should not have the goal of only improving the vaccination rates through improved physical accessibility. Even within public health, this is more important for immunization to prevent especially infectious diseases (36). The condition for privatization to resolve inequality is when clarification of the target population and alleviation of healthcare utilization inequalities among such a population are set as the major goals (34). For example, contracting out was able to alleviate vaccination inequality in Cambodia, where the contract stipulated equality as one of the roles of the service provider (37). In this context, contracting out healthcare services could go beyond cost saving and enhanced efficiency to ask questions about the mission and value of public health (12). The value of public health can be found in not only health promotion and prevention among individuals and populations, but also equitable and just distribution. Therefore, a key responsibility of public health is to simultaneously consider both the structural factors of diseases and the social determinants of health. Of course, it should be based on the understanding of the mechanisms behind the utilization of public health services by the target population. While the authorities identify demands, procurement, allocation and reapportion of influenza vaccines, managing leftovers, and controlling hazards, they did not address the oversight of the quality of the NIP itself. In other words, there has been no responsible stewardship on quality assurance for alleviating vaccination inequality among the elderly in the NIP. Thus, the quality control system for contracting out the NIP should be established.

The present study had some limitations. First, information about where people were vaccinated for influenza (public or private medical institution) was unavailable; thus, the study was limited to identifying the total impact of the vaccination rate. For example, if the proportions of vaccinations in the public and private sectors after contracting out were measured directly and contributing factors were identified to determine where changes in vaccination rates based on gender or insurance type occurred, more specific conclusions could have been drawn. Second, while barriers to physical and cost accessibility were partially alleviated through contracting out, information about other factors that could influence improvement in vaccination rates, such as improvement in information accessibility or acceptance, was insufficient. Therefore, the mechanism could only be estimated. Third, the study did not analyze whether the effects of contracting out appear differently according to the distribution of public/private medical resources within a region. Additional analysis that links data regarding the distribution of medical resources is warranted in the future. Fourth, the analyses did not consider potential time-varying confounders other than the incidence of influenza in the (t-1) year owing to small number of time points in the model. Finally, although cost effectiveness is one of the main reasons to contract out NIP, we could not access the information about the government expenditure on this program and the result of policy evaluation including its cost-effectiveness. Future studies should focus on the economic evaluation of the new strategy of NIP.

The number of public healthcare institutions, including public health centers is small; thus, offering vaccinations through only public institutions would limit availability and physical accessibility within the region. Therefore, increasing the number of institutions that provide such a service through contracting out could be viewed as an easy approach. However, the present study did not find any improvement that exceeded the existing increasing trend in the vaccination rate, nor did it find improvement in socioeconomic inequality. Nonetheless, a significant increase was found among poor elderly women who are medical aid recipients. This is suspected to be the effect of improved cost accessibility as a result of a waiver of out-of-pocket costs.

When contracting out, a mandate for alleviating health inequality was not specifically imposed on the private institutions. Meanwhile, public institutions are downsizing vaccination services in response to the expansion of contracting out. Furthermore, demand is also decreasing with the decreasing population size in non-urban regions. This is causing greater inequality in the distribution of private medical institutions between regions. Therefore, privatization of vaccination services is highly likely to worsen such inequality. Outcomes from contracting out, which was attempted to address insufficient public health infrastructure, need to be reassessed. In particular, it is necessary to monitor the long-term effects related to health inequality.

## Data Availability Statement

Publicly available datasets were analyzed in this study. This data can be found here: https://chs.kdca.go.kr/chs/rdr/rdrInfoProcessMain.do.

## Ethics Statement

The studies involving human participants were reviewed and approved by Institutional Review Board of Konyang University. Written informed consent for participation was not required for this study in accordance with the national legislation and the institutional requirements.

## Author Contributions

HC and DM conceptualized the study. HC designed the analysis plan, performed the formal analysis, and created the figures. DM, SK, M-HK, and DJ contributed to writing the manuscript. All authors approved the final version for submission.

## Funding

The study was supported by a grant from the National Research Foundation of Korea (NRF) funded by the Korean Government (Ministry of Science and ICT: MSIT; No. NRF2021R1F1A105102111) (HC).

## Conflict of Interest

The authors declare that the research was conducted in the absence of any commercial or financial relationships that could be construed as a potential conflict of interest.

## Publisher's Note

All claims expressed in this article are solely those of the authors and do not necessarily represent those of their affiliated organizations, or those of the publisher, the editors and the reviewers. Any product that may be evaluated in this article, or claim that may be made by its manufacturer, is not guaranteed or endorsed by the publisher.
